# An interesting interface: Ingenious improvisation meets troubleshooting, lessons learned and thoughts to be shared

**DOI:** 10.1016/j.ipej.2026.01.008

**Published:** 2026-01-12

**Authors:** Anindya Ghosh, Chenni S. Sriram, Deep Chandh Raja

**Affiliations:** a Department of Cardiac Electrophysiology and Pacing, Arrhythmia Heart Failure Academy, The Madras Medical Mission, Chennai, Tamil Nadu, India; b Division of Cardiology, Sub-section of Electrophysiology, Children's Hospital of Michigan and Detroit Medical Center, Detroit, MI, USA; c Department of Cardiac Electrophysiology and Pacing, Kauvery Hospital, Chennai, India

**Keywords:** Atrial fibrillation, Pacemaker mediated tachycardia, T wave oversensing, Biventricular pacemaker

## 1 Case presentation

A 50-year-old gentleman with permanent atrial fibrillation and fast ventricular response with ventricular dysfunction and recurrent heart failure hospitalizations underwent atrioventricular (AV) node ablation. Following this, biventricular pacing was performed with one lead (Abbott 1258 Quickflex M) in the posterolateral coronary sinus (CS) tributary connected to the atrial port and another in the right ventricular (RV) apex connected to the ventricular port of a dual chamber pulse generator (Abbott Endurity™ Core 2152).

The device was programmed to DDD mode with an ultrashort AV delay (sensed/paced) of 25 ms. The lower rate was set 70 beats per minute (bpm) and upper tracking rate (UTR) was 130 bpm. The sensitivity on the atrial and ventricular channels were 0.5 mV and 2.0 mV respectively. In addition, automatic mode switch and premature ventricular contraction (PVC) response were turned “OFF”, and post ventricular atrial blanking (PVAB) was kept at 120 ms. The device was configured to rate-responsive post-ventricular atrial refractory period (PVARP) with baseline of 275 ms and minimum of 125 ms.

During evaluation, fluoroscopy demonstrated unchanged lead position (Supplementary Fig. 1). What is the probable mechanistic explanation for the electrocardiograms showcased in Fig. 1A, Fig. 1B?

**Fig. 1A:**
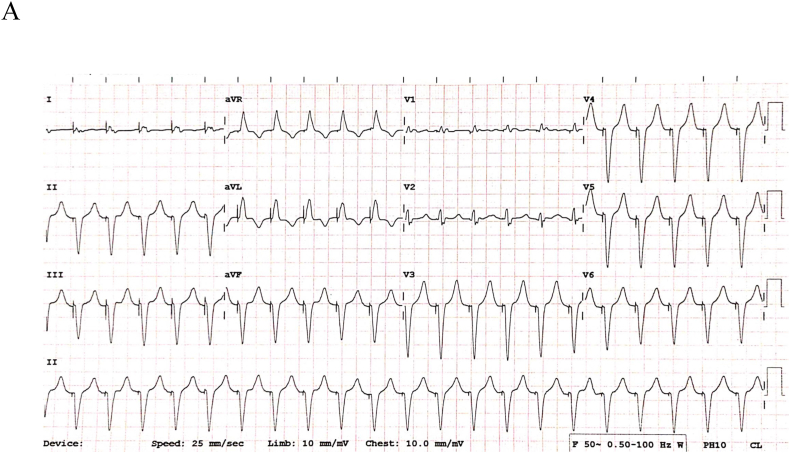
Presenting 12-lead electrocardiogram during palpitat1ons.

**Fig. 1B:**
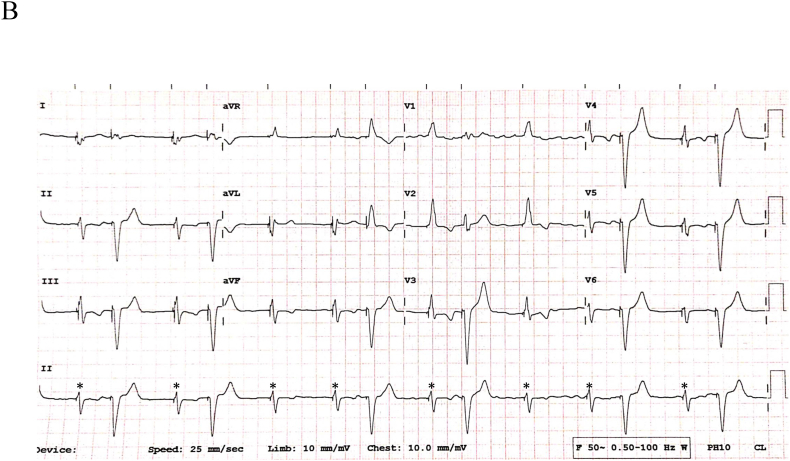
12-lead ECG at another instance during the observation period.

## 2 Discussion

Preliminary analysis of Fig. 1A reveals a regular monomorphic wide complex tachycardia at the UTR of 130 bpm. Pacing artifacts precede all QRS complexes with morphology consistent with RV apical pacing. P waves are not discernible. Fig. 1B shows underlying atrial fibrillation with two different paced QRS morphologies. The narrower QRS (asterisk ∗; 120 ms) represents biventricular pacing (corresponding two distinct pacing spikes seen in lead III/V3) with its characteristic QRS signature. The broader QRS complex is indicative of RV apical pacing only (matches Fig. 1A). In this context, it is likely that the patient was in persistent atrial fibrillation (even in Fig. 1A). LV-alone paced morphology is presented in Supplementary Fig. 2.

In a conventional dual chamber pacemaker with atrial and RV leads, the differential diagnoses of Fig. 1A would include, i) pacemaker mediated tachycardia (PMT), ii) coincident sinus/atrial tachycardia with rate approximating the UTR that is tracked by the ventricular channel and, iii) a blanked flutter (atrial rate of 260 bpm) undetected by the pacemaker algorithm. T wave oversensing on the atrial channel outside the post-ventricular atrial refractory period (PVARP) is a remote possibility and mechanistically similar to PMT.

However, our tracing should be analyzed in the context of the associated ‘MacGyvering’. Here, the non-dislodged LV lead represents the atrial channel and any sensed events outside the PVARP would be annotated as atrial-sensed (***As***) with all its ensuing consequences. In the absence of an intrinsic ventricular rhythm, any such ***As*** events can only be due to far-field atrial sensing on the LV lead or secondary to paced ventricular depolarization (QRS)/repolarization (T wave). In the setting of persistent atrial fibrillation, far-field atrial sensing cannot explain the regular tracking as seen in Fig. 1A. Also, the LV lead is unlikely to sense the paced QRS outside the PVARP. Theoretically, this is only feasible if there is a setup for loss of LV capture with continued RV pacing and substantial inter-ventricular conduction delay to the LV lead.

However, the latter phenomenon of T wave oversensing (TWOS) on the LV lead (atrial channel) outside the PVARP is far more plausible here for reasons articulated below. In normal dual chamber pacemakers, the far-field T waves are algorithmically rejected (not annotated as atrial events) by the atrial channel due to its lower frequency/slew rates (Ref). This paradigm may not hold true in this singular instance for obvious reasons. Once TWOS is annotated as ***As***, LV (atrial channel) pacing is automatically inhibited. The device switches to a tracking mode (***Ap***-***Vp*** to ***As***-***Vp***) commensurate with extension of the sensed AV delay so as to avoid violation of UTR. PMT ensues as long as TWOS is persistent. Any proprietary algorithms to terminate such a PMT will provide only temporary reprieve.

**Fig. 2:**
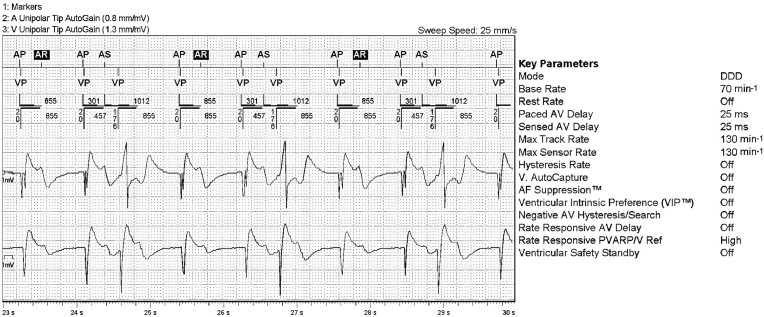
Device electrograms corresponding to Fig. 1B (Refer to text for details).

Intermittent TWOS by the LV lead (atrial channel) is also a plausible explanation for Fig. 1B. During biventricular pacing, there is narrower QRS. With onset of TWOS (annotated ***As***), LV pacing is inhibited with consequent tracking and RV only pacing. This hypothesis was confirmed on evaluation of device electrograms (Fig. 2). The etiology of intermittent TWOS was secondary to its critical timing just within (annotated as sensed refractory event or ***AR***) or outside PVARP (***As***).

One particular caveat noted on Fig. 1A warrants further scrutiny. There are 21 paced QRS complexes noted without any nominally programmed proprietary PMT termination algorithm (Auto detect™) at play. The latter is typically triggered after 8 beats at the UTR. There is ensuing alteration of the AV delay (shortened by 50 ms of ***As-Vp*** >100 ms or extended by 50 ms if ***As-Vp*** <100 ms). This is followed by confirmation of PMT if Vp-As interval is deemed regular (≤16 ms). Upon this, ***Vp*** is suspended for 1 cycle followed by atrial pacing delivered 330 ms after the detected P wave (similar to PVARP extension followed by timed atrial pacing) [1].

In the absence of any corresponding device electrograms/alerts, the authors can only speculate as follows. It is known that if the algorithm fails to confirm PMT at first instance, further detection is postponed for 256 beats and henceforth for a period of 5 minutes at all subsequent confirmation failures. The simplest explanation for Fig. 1A is PMT secondary to consecutive TWOS (all ***As*** and no ***AR*** events) but failure of confirmation (due to exaggerated variations in ***Vp-As*** intervals >16 ms). Other complex explanations are possible but are not discussed for the sake of brevity.

In retrospect, this iatrogenic PMT was the result of a programming glitch and is an avoidable entity. The over sensed T waves on the LV lead (atrial channel) were just above the sensitivity threshold of 0.5 mV. Decreasing the sensitivity on the atrial channel (in this case the LV sensitivity) to 2.0 mV without changing other parameters resolved the problem with subsequent assured biventricular pacing. There are few other alternatives to this. One can consider changing to a non-tracking and atrial pace-only mode i.e. DVI or more commonly the non-tracking dual chamber mode DDI. In summary, the possibility of this iatrogenic PMT should be kept in mind for the pacing configuration as described in this case. Appropriately programming parameters/modes of sensing and pacing can be adopted preemptively to avoid such an occurrence. To the best of our knowledge, this is the first report of its kind in published literature and stresses the need for a basic and in-depth understanding of pacemaker timing cycles.

## Patient consent

Informed patient consent was taken for this manuscript preparation.

## Ethical statement

Ethics committee approval was taken for this case report.

## Declaration of competing interest

The authors declare that they have no known competing financial interests or personal relationships that could have appeared to influence the work reported in this paper. The authors did not receive any funding for the preparation of this manuscript.
