# Supplementary figures and images for: An interesting interface: Ingenious improvisation meets troubleshooting, lessons learned and thoughts to be shared
Source: Indian Pacing Electrophysiol J. 2026 Jan 12;26(1):50–2. doi: 10.1016/j.ipej.2026.01.008 (PMC12958033; doi:10.1016/j.ipej.2026.01.008)

## Slide 1
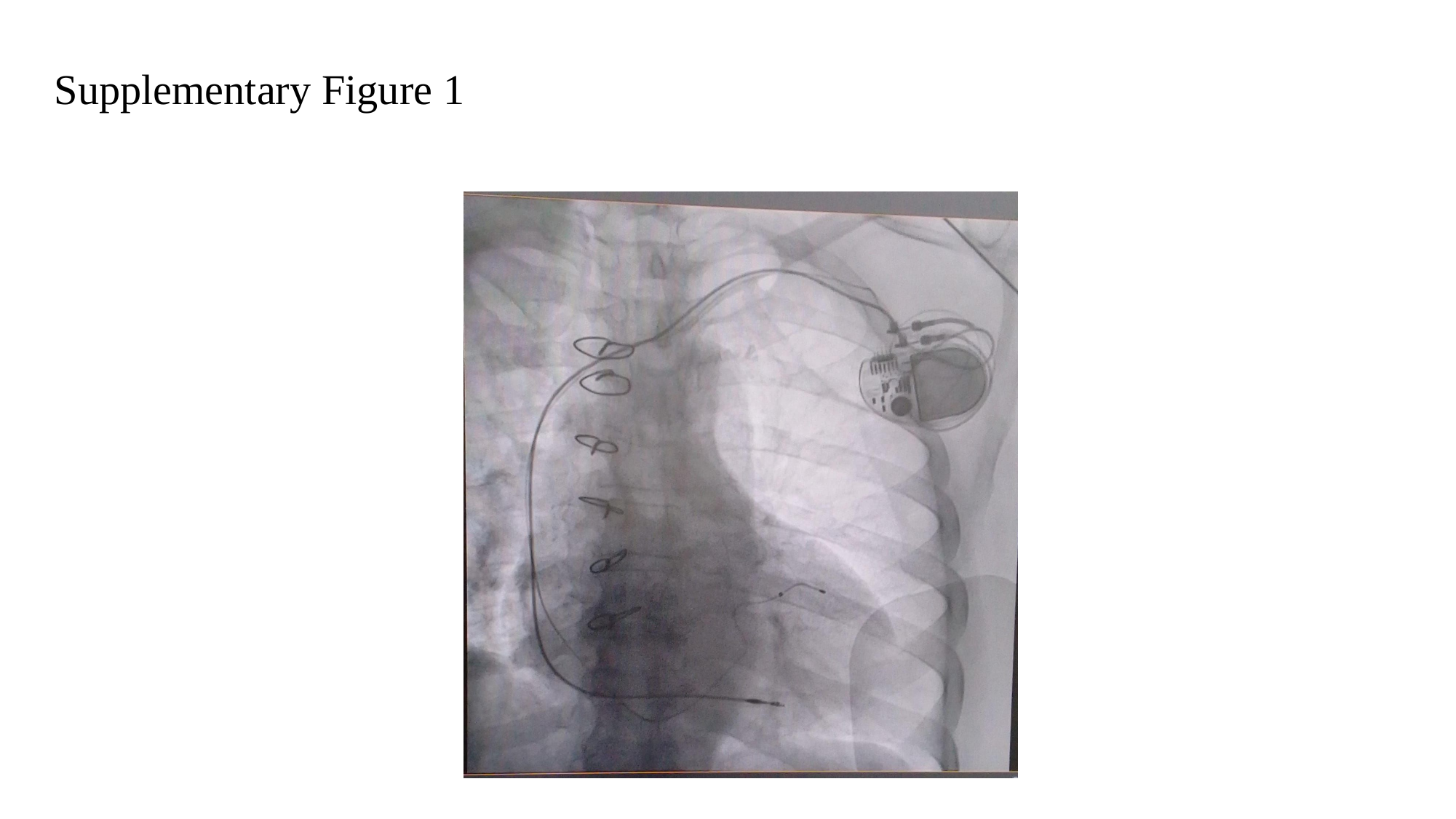

Supplementary Figure 1

## Slide 2
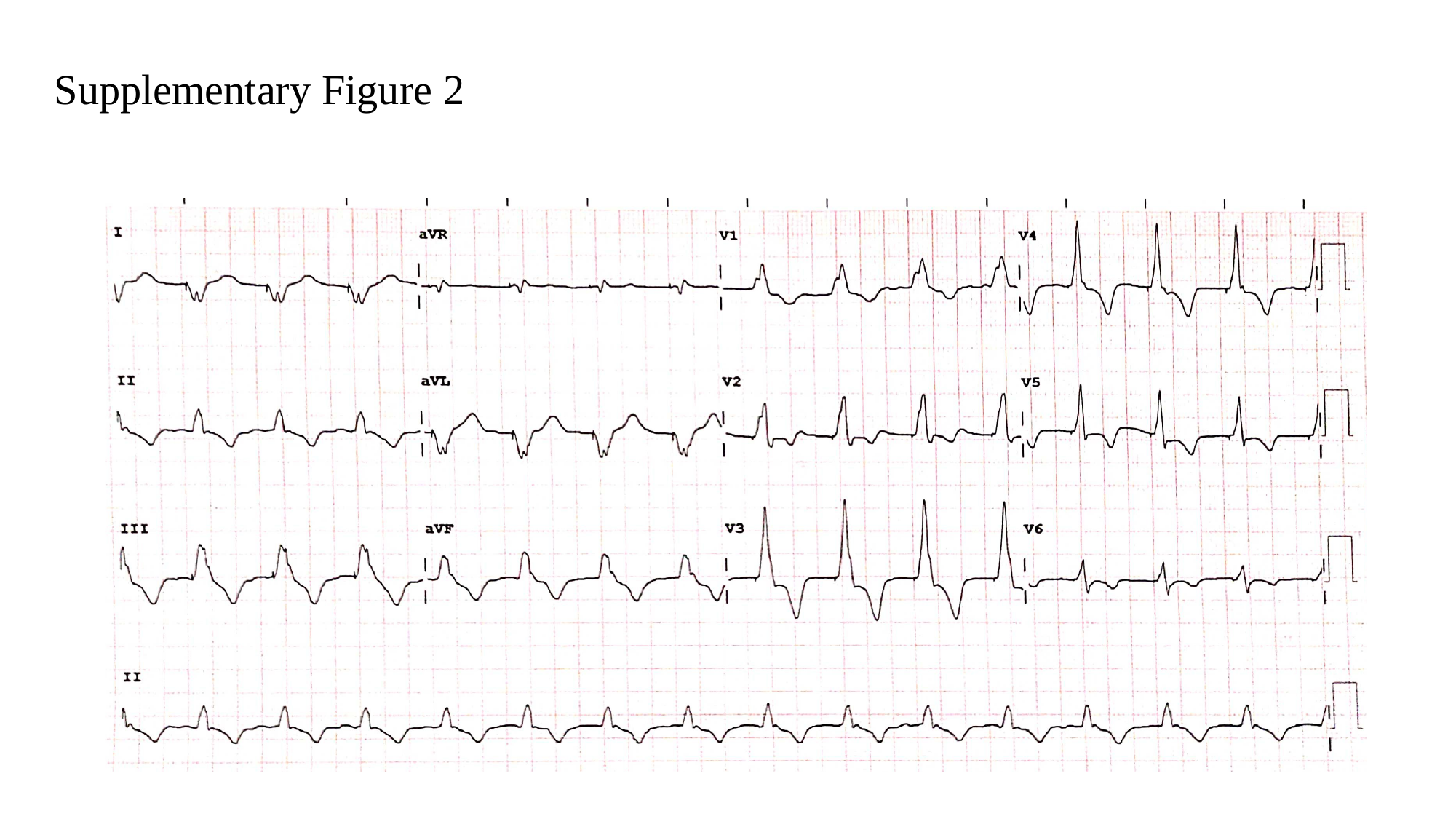

Supplementary Figure 2

Supplement: Multimedia component 1 [file mmc1.pptx]
